# Genotyping horse epithelial cells from fecal matter by isolation of polymerase chain reaction products

**DOI:** 10.3325/cmj.2017.58.239

**Published:** 2017-06

**Authors:** Pero Dimsoski

**Affiliations:** 1 Department of Chemistry and Biochemistry, Florida International University, Miami, USA

## Abstract

**Aim:**

To show that application of the polymerase chain reaction (PCR) method modified for amplification of a low-copy number DNA samples, ie, the isolation of PCR products (IPCRp), would represent improvement in obtaining genotypes from a fecal DNA compared with previously used genotyping methods.

**Methods:**

The DNA from the horse fecal matter was extracted by modified Qiagen DNA Stool Mini Kit protocol. Following the extraction, the DNA genotypes from fecal samples were obtained by the most powerful PCR amplification method, the IPCRp. The IPCRp-based multiplex kit amplified biotin-labeled strands were captured on streptavidin-coated plates, where everything but the dye-labeled target sequence was washed, eliminating all the background noise, released, and run on a genotyping instrument in a single-strand configuration.

**Results:**

The IPCRp-based multiplex kit (6 loci) revealed equine DNA full genotype profiles, ie, appearance of all six loci, when sampled from fresh feces in 87% of the samples and partial genotype profile (appearance of one to five loci) in 13% of the samples, for a total of 100% genotyping success rate.

**Conclusion:**

These results indicate that the IPCRp amplification method, coupled with the Qiagen DNA Stool Mini Kit extraction can maximize the likelihood of obtaining horse DNA genotypes from fecal samples.

The fecal matter is a complex mixture of microbes, digested food by-products, enzymes, and bile salts, which all together adversely influence successful DNA genotyping due to contamination, degradation, and inhibition issues (1). Additionally, it has been shown that a DNA from a non-invasive samples is often of a low quantity and quality contributing toward the low success rate in obtaining genetic profiles (2,3). Therefore, the aim of this study was to show that the application of the polymerase chain reaction (PCR) method, modified for the amplification of a low-copy number DNA samples, the Isolation of PCR products (IPCRp) (4), would represent improvement in obtaining genotypes from a fecal DNA compared with previously used genotyping methods.

## MATERIAL AND METHODS

Fecal matter from five domestic horses of known origin was collected within three hours of defecation and stored at 4°C until used for extraction in triplicates, which was performed within seven days of collection. Reference hair samples from the same horses were used as positive controls and were extracted, profiled, and compared to the fecal extractions.

Because equine gut epithelial cells are often more abundant on the outside of fecal matter, the outer layer of stool samples were either scraped or swabbed prior to DNA extraction (1,5). For scraped samples, sterile scalpels were used to sheer off approximately 220 mg from the outer fecal layer. For swabbed samples, sterile cotton swabs were used to wipe the outer fecal layer while rotating the swab to ensure maximum recovery of cells. Both scraped and swabbed samples were stored in 2-mL tubes until extracted.

### DNA extraction

Fragment analysis results using the Qiagen QIAmp® DNA Stool Mini Kit manufacturer’s suggested protocol were compared to the Qiagen stool kit protocol with modifications. Optimization of the Qiagen QIAmp® DNA Stool Mini Kit (Qiagen, Valencia, California, USA) was performed in which replicate fecal samples were extracted by either the manufacturer’s suggested protocol for the DNA analysis or modifications to the Qiagen stool kit protocol (6-8). The modifications included the combination of swabbing the fecal matter; digesting the samples overnight in 1.0 mL of Buffer ASL with 25 μL of proteinase K in a thermomixer (Eppendorf, Hamburg, Germany) at 55°C and 300 RPM; adding half of an inhibit EX tablet instead of a full tablet; not digesting proteinase K in Buffer AL; adding 1 μL of carrier RNA prior to 70°C incubation in order to enhance the binding of DNA to the Qiagen column; eluting in 50 μL of diethyl pyrocarbonate (DEPC)-treated water at room temperature; and incubating at room temperature for five minutes prior to the final spin down.

### PCR amplification

Equine specific primers for the short tandem repeat (STR) markers were used to assemble both, 6-plex PCR kit and IPCRp-6plex kit (Table 1).

**Table 1:** Primer sequences for equine 6-plex polymerase chain reaction (PCR)*

Locus	Dye	Size range (bp)	Primer sequence (5′-3′)
VHL20	6-FAM	83-102	F: CAAGTCCTCTTACTTGAAGACTAG R: AACTCAGGGAGAATCTTCCTCAG
HTG4	6-FAM	116-137	F: CTATCTCAGTCTTGATTGCAGGAC R: CTCCCTCCCTCCCTCTGTTCTC
HTG6	VIC	74-103	F: GTTCACTGAATGTCAAATTCTGCT R: CCTGCTTGGAGGCTGTGATAAGAT
HMS6	VIC	154-170	F: GAAGCTGCCAGTATTCAACCATTG R: CTCCATCTTGTGAAGTGTAACTCA
HTG7	NED	114-128	F: CCTGAAGCAGAACATCCCTCCTTG R: ATAAAGTGTCTGGGCAGAGCTGCT
HMS3	NED	146-170	F: CCAACTCTTTGTCACATAACAAGA R: CCATCCTCACTTTTTCACTTTGTT

* Fluorochromes are located at the 5′-end of the forward primer. Biotin labeled 5′-end of the reverse primer for isolation of PCR products (IPCRp)-6plex kit were used.

Equine specific primers for STR markers VHL20, HTG4, HTG6, HMS7, HTG7 and HMS3 were mixed in pre-determined quantities to make an in-house 6-plex PCR kit for research use only (9-12). The in-house 6-plex PCR kit was compared with the IPCRp-multiplexing kit, also assembled in-house for research use only, that was exactly the same as the 6-Plex PCR kit, with the only difference being biotin-labeled 5′-end of the forward primers while the fluorescent dye was conventionally located at the 5′-end of the reverse primer (4). An amplification volume of 15 μL was used containing 2.5 μL of StockMarks® PCR buffer, 4 μL of StockMarks® dNTPs, 4 μL of standard 6-plex primer mix or IPCRp 6-plex primer mix, and 0.5 μL of AmpliTaq Gold® polymerase, as per the StockMarks® for horses protocol (Applied Biosystems, Foster City, CA). The PCR was carried out on the GeneAmp® PCR system 9700 thermal cycler in 9600 emulation mode under the following conditions: 95°C for 10 minutes; 30 cycles of 95°C for 30 seconds, 60°C for 30 seconds, and 72°C for 60 seconds; with a final hold at 72°C for 60 minutes (13). After the PCR, 1 μL of each equine DNA product from the standard 6-plex kit (n = 9 per horse) was then separated on the ABI 310 Genetic Analyzer (Applied Biosystems), while 1 μL of equine DNA product from the IPCRp 6-plex kit (n = 9 per horse) was captured onto a Pierce® streptavidin-coated High Binding Capacity (HBC) clear 96-well plate with SuperBlock® blocking buffer (Thermo Scientific, Rockford, IL). The IPCRp amplification was performed on two different domestic horses (Horse #1 and Horse #2) as a comparison.

After the amplification with the IPCRp-multiplex kit, the products of the reaction were processed according to described protocol (4) with few modifications. A 10 μL of 10 × sodium chloride-sodium citrate (SSC) buffer, along with 1 μL of IPCRp amplified product were added to wells on streptavidin-coated plates and incubated for 15 minutes at room temperature and at 350 RPM on the thermomixer. The supernatant was pipetted out and 250 μL of DEPC water was used to wash wells. Wells were washed an additional two times with 250 μL of DEPC water to remove all excess enzymes, dNTPs, and unincorporated primers. After wells had been removed of all liquid, 11.5 μL of HiDiTM formamide mixed with 0.5 μL of GeneScan LIZ600TM internal size standard (Applied Biosystems) was added to denature and release the dye-labeled DNA strand followed by transferring the HiDi mixture to 0.5 μL tubes for capillary electrophoresis and fragment analysis. The post-amplification handling of IPCRp samples added extra 20 minutes, compared to the standard PCR protocols.

To show maximum clarity of equine DNA profiles following the IPCRp step, tests were conducted to determine optimal amount of the IPCRp product to add to the streptavidin plate. For comparison, 1 μL, 2 μL, and 5 μL of select PCR samples were used and obtained genotypes were compared. All other capture and purification steps remained the same as previously described.

### Genotyping and analysis

The DNA fragments were separated on the ABI 310 Genetic Analyzer using module DS33, filter G5v2, and GeneScan LIZ600TM internal size standard and were analyzed using GeneMapper® 3.7 software (Applied Biosystems). Successful amplifications were determined when amplified allele matched the expected fragment length of the corresponding positive hair sample using a threshold of 50 RFU. Two-tailed *t* tests with a 95% confidence interval (CI) and one-way ANOVA analyses were used to analyze corresponding peak heights and expected amplified alleles for all optimization comparisons.

Once optimization was completed and fecal extraction and PCR amplification techniques were determined, the methods were applied to all five domestic horses in triplicate (n = 15) to determine if full equine DNA profiles from fresh fecal samples could be obtained and matched the positive control for which the DNA was extracted from the hair samples. Additionally, the methods were applied to determine the degree to which a sample could be compromised by the age of sample. One domestic horse (Horse #2), previously profiled, was used for this study. Fecal samples were left in its natural environment up to eight days after defecation and were collected on even days (ie, Days 0, 2, 4, 6, 8) and ran in triplicate (n = 15) to determine if the horse genotype could still be obtained.

## RESULTS

**Figure 1:**
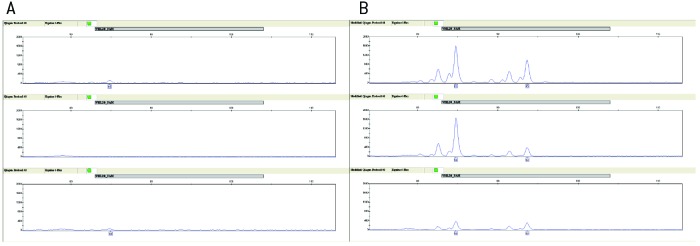
Electropherograms of equine fecal DNA profiles at locus VHL20 using (a) the manufacturer’s suggested protocol for the Qiagen QIAmp® DNA Stool Mini Kit and (b) modified Qiagen protocol (6,7).

Peak heights obtained by Qiagen stool kit modified protocol improved the genotyping results, as measured by the higher peak heights of electropherograms, probably due to improved DNA yield, compared with the peak heights obtained by amplification of DNA extracted with Qiagen kit using manufacturer’s suggested standard protocol (Figure 1a and 1b).

The comparison between the standard 6-plex and the IPCRp 6-plex for both Horse #1 and Horse #2, for the number of amplified alleles per DNA profile was compared to the expected number of amplified alleles and averaged per triplicate PCR to obtain the percent of expected alleles amplified (Table 2).

**Table 2:** Average number of amplified alleles present from fecal matter for both the standard polymerase chain reaction (PCR) 6-plex kit and the isolation of PCR products (IPCRp) 6-plex kit compared to expected number of alleles for Horse #1 and Horse #2.

Amplification method	Sample	Amplified number of alleles	Average amplified number of alleles	Percentage of expected alleles amplified	Amplification method	Amplified number of alleles	Average amplified number of alleles	Percentage of expected alleles amplified
**Horse #1**								
Standard 6-Plex Kit	1.1	10	10	83	IPCRp 6-Plex Kit	11	11	92
	1.2	10				11		
	1.3	10				11		
	2.1	8	6.33	52		7	7	58
	2.2	5				9		
	2.3	6				5		
	3.1	5	3	25		3	3.67	31
	3.2	3				3		
	3.3	1				5		
**Horse #2**								
Standard 6-Plex Kit	1.1	10	10	83	IPCRp 6-Plex Kit	12	12	100
	1.2	11				12		
	1.3	9				12		
	2.1	12	12	100		12	11.7	97
	2.2	12				12		
	2.3	12				11		
	3.1	12	12	100		12	12	100
	3.2	12				12		
	3.3	12				12		

**Figure 2:**
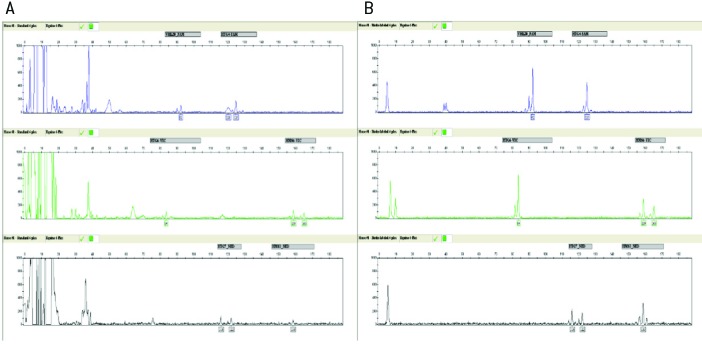
Equine DNA profiles from Horse #2 comparing (a) 1 μL of standard polymerase chain reaction (PCR) 6-plex kit and (b) 1 μL of 6-plex kit for the isolation of PCR products (IPCRp) where the IPCRp 6-plex kit showed a dramatic decrease in primer fronts, dye blobs, and back-ground noise while an increase in signal strength.

A lack of an amplified allele meant complete allele drop out. For Horse #1, the average peak height for all alleles increased by 1.95 when using the IPCRp 6-plex compared to the standard 6-plex. Likewise, for Horse #2, the average peak height increased by 1.65, leading to an overall increase of 1.8 for all 36 samples combined when using the IPCRp method compared to the standard 6-plex. The IPCRp not only increased the overall percentage of the expected alleles amplified, but also increased signal strength and dramatically reduced background noise while eliminating dye blobs and unincorporated primers, increasing equine profile clarity for fecal samples (Figure 2).

**Figure 3:**
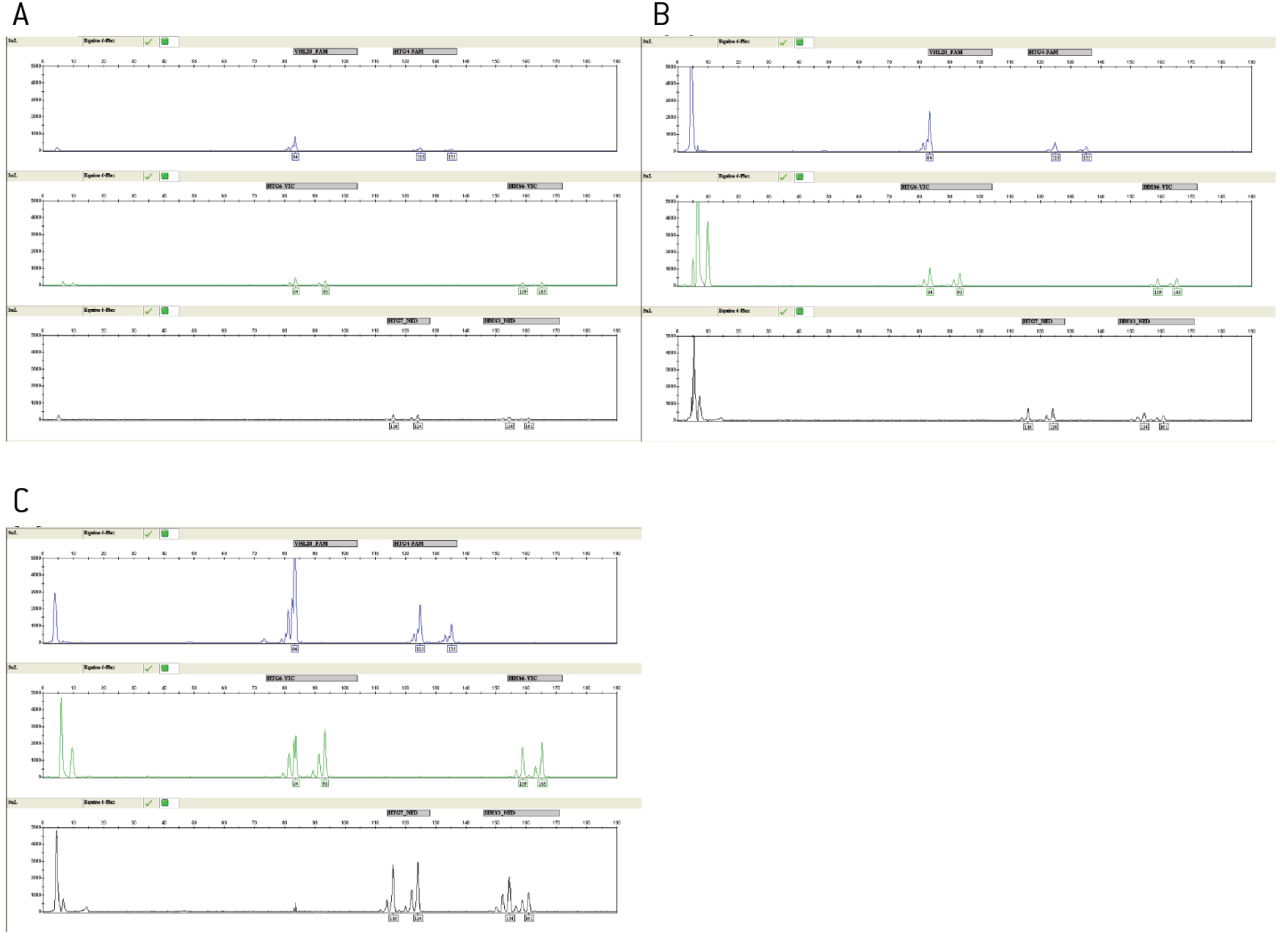
Equine DNA profiles comparing the isolation of polymerase chain reaction products (IPCRp) method where (a) 1 μL of polymerase chain reaction (PCR) product, (b) 2 μL of PCR product, or (c) 5 μL of PCR product was added to the streptavidin-coated plate for capture and separation.

Also, the volume of the IPCRp product added to the streptavidin-coated plate was subsequently adjusted to determine if the increased product volume led to a greater number of full profiles compared to partial profiles as well as improved signal strength for called alleles. When 1 μL, 2 μL, and 5 μL PCR product volumes were compared, signal strength greatly improved as the input amount increased (Figure 3).

Alleles that were not visible with 1 μL of IPCRp product could be seen and analyzed with 5 μL of IPCRp product input onto the streptavidin-coated plate, showing that an increase in the product volume improved analyses of the DNA profiles without the increase in stutter (*P* < 0.05).

### Application

**Figure 4:**
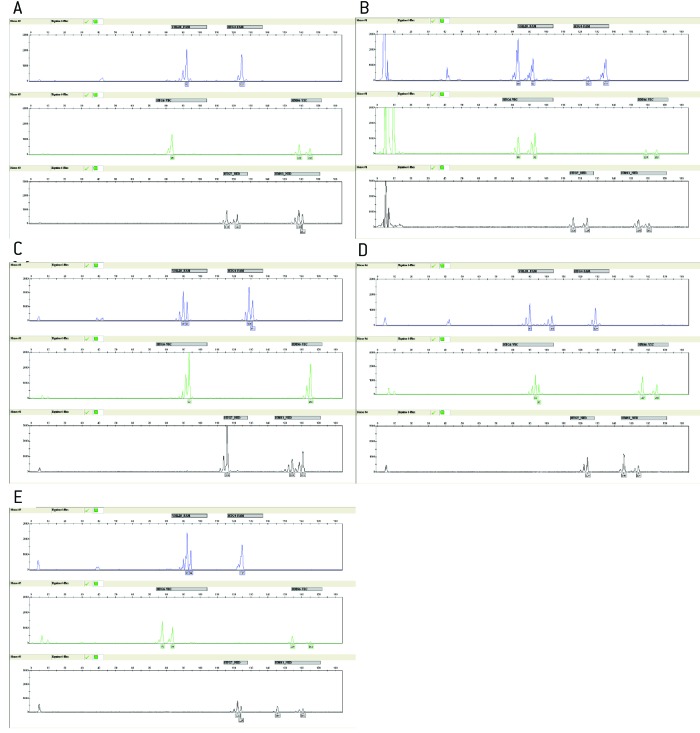
Equine DNA profiles obtained from fecal matter using the isolation of polymerase chain reaction products (IPCRp) method for (a) Horse #1, (b) Horse #2, (c) Horse #3, (d) Horse #4, and (e) Horse #5.

Based on the optimized results, it was determined that the Qiagen QIAmp® DNA Stool Mini Kit protocol with select modifications, along with the incorporation of the extremely powerful IPCRp amplification/genotyping method worked best when differentially extracting equine gut epithelial cells from fecal matter to obtain a DNA profile. When applying this method to fecal samples from five different domestic horses, at least one full DNA profile (six loci) could be obtained for every horse (Figure 4). In total, successful full profiles were obtained in 13 of 15 samples while partial profiles (one to five loci) were obtained in the other two samples. This demonstrated an 87% success rate in acquiring full equine DNA profiles from fresh fecal samples.

**Figure 5:**
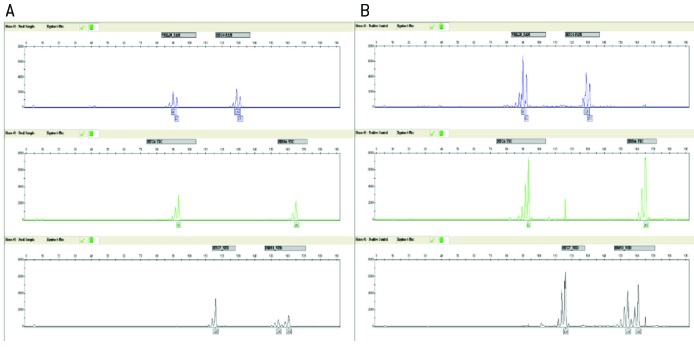
Equine DNA profiles from Horse #3 comparing fecal sample amplified by the isolation of polymerase chain reaction products (IPCRp) method (a) and hair sample (b, positive control).

Additionally, equine DNA profiles from fecal samples were identical to positive control hair samples from all five horses showing extraction and purification methods to be reliable (Figure 5).

**Figure 6:**
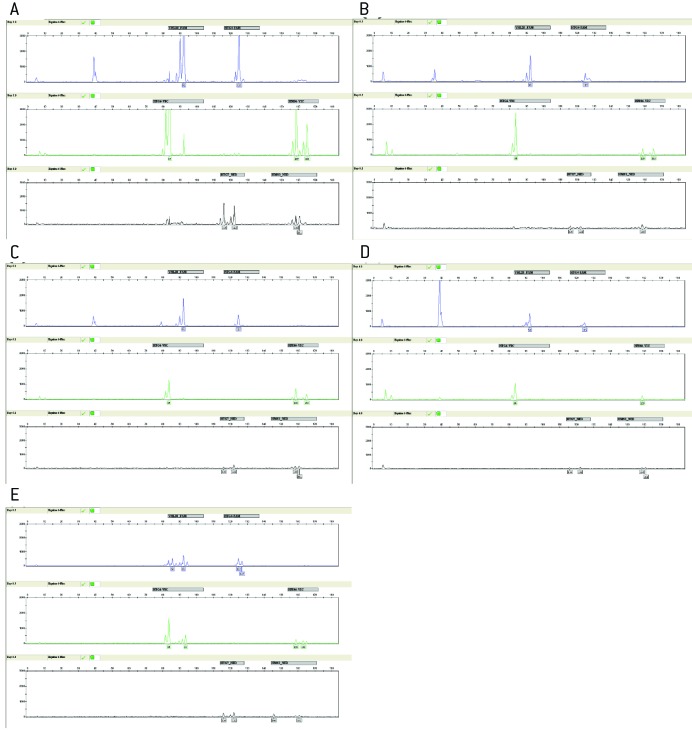
Equine DNA profiles from Horse #2 using the isolation of polymerase chain reaction products (IPCRp) method for aged fecal samples after 0 Days (a), 2 Days (b), 4 Days c), 6 Days (d), and 8 Days (e).

Analysis of the aged samples from Horse #2 collected for eight days after defecation showed that full equine DNA profiles from feces could be obtained in 67% of samples after two days, 33% of samples after four days, and 0% of samples after six and eight days. The number of amplified alleles per collection day was compared to the expected number of amplified alleles and averaged per triplicate sample to obtain the percent of expected alleles amplified. Signal strength decreased the longer the fecal matter remained in the outside environment (Figure 6). After four days, DNA profiles became more unreliable at each marker through the decrease in signal quality and profile clarity and through the appearance of false alleles.

## DISCUSSION

For the fresh fecal samples from five domestic horses IPCRp method improved the likelihood of obtaining full equine DNA profiles. Overall, successful profiles, where all expected allele peaks amplified, were obtained in 87% of samples from fresh fecal matter. In previous research, successful fecal DNA profiles were obtained in 73% of brown bear samples (14), 53% of wolf samples (15), and 21% of tiger samples (16). The IPCRp amplification method virtually eliminated the stutter, including the dye-labeled unincorporated primers that are usually visible in the 0-100 bp ranges of the capillary electrophoresis generated electropherograms. Previous studies have applied variations of the streptavidin-biotin binding complex to their work in combination with DNA complimentary probes (3,17). However, in the IPCRp method the probe was the other DNA strand generated during the PCR reaction (4). The design where the probe is generated during the amplification assured the maximum production of the dye-labeled sequence of interest and maximized the capture efficiency of the targeted, dye-labeled DNA. Obtaining visibility for previously invisible alleles by increasing a quantity of the amplified product is adding an extraordinary feature to the capillary-based fragment analysis instruments such as ABI 310, very similar to the volume dial on the stereo. This is another unique characteristic of the IPCRp amplification reaction. Previously, with other PCR-based methods, increasing the volume of the PCR product into fragment analysis instrument would also increase the background noise, impeding the genotyping. However, too much product could lead to overloading, reaching the limit of the maximum detection of the ABI310 Genotyper. Another interesting aspect of the IPCRp is showing alleles that did not appear with classical PCR amplification. It is an established fact that the stochastic processes, especially when using the degraded, low-copy number DNA, can influence the scoring of the state of a locus (18). This effect should be of a major concern because not being able to distinguish between heterozygote and homozygote state would produce not only erroneous results but erroneous conclusions as well in identification or genetic studies. Biologically, there is nothing that would suggest a copy differential preference of the restriction enzymes, so, most likely, observed stochastic effects are part of the amplification/genotyping process itself. However, with IPCRp, this stochastic error could be minimized due to possibility of using the whole volume of the DNA available for both, amplification and detection. Therefore, lowering the recommended template quantities when degraded DNA is amplified (19) should be considered. In addition, with the IPCRp, there is no need of additional PCR cycles, a common practice to amplify a low copy number DNA, which together with a a washing step eliminates stutter completely, therefore, eliminating one of the three factors (20) adding to stochastic effects when a low template DNA is amplified.

**Figure 7:**
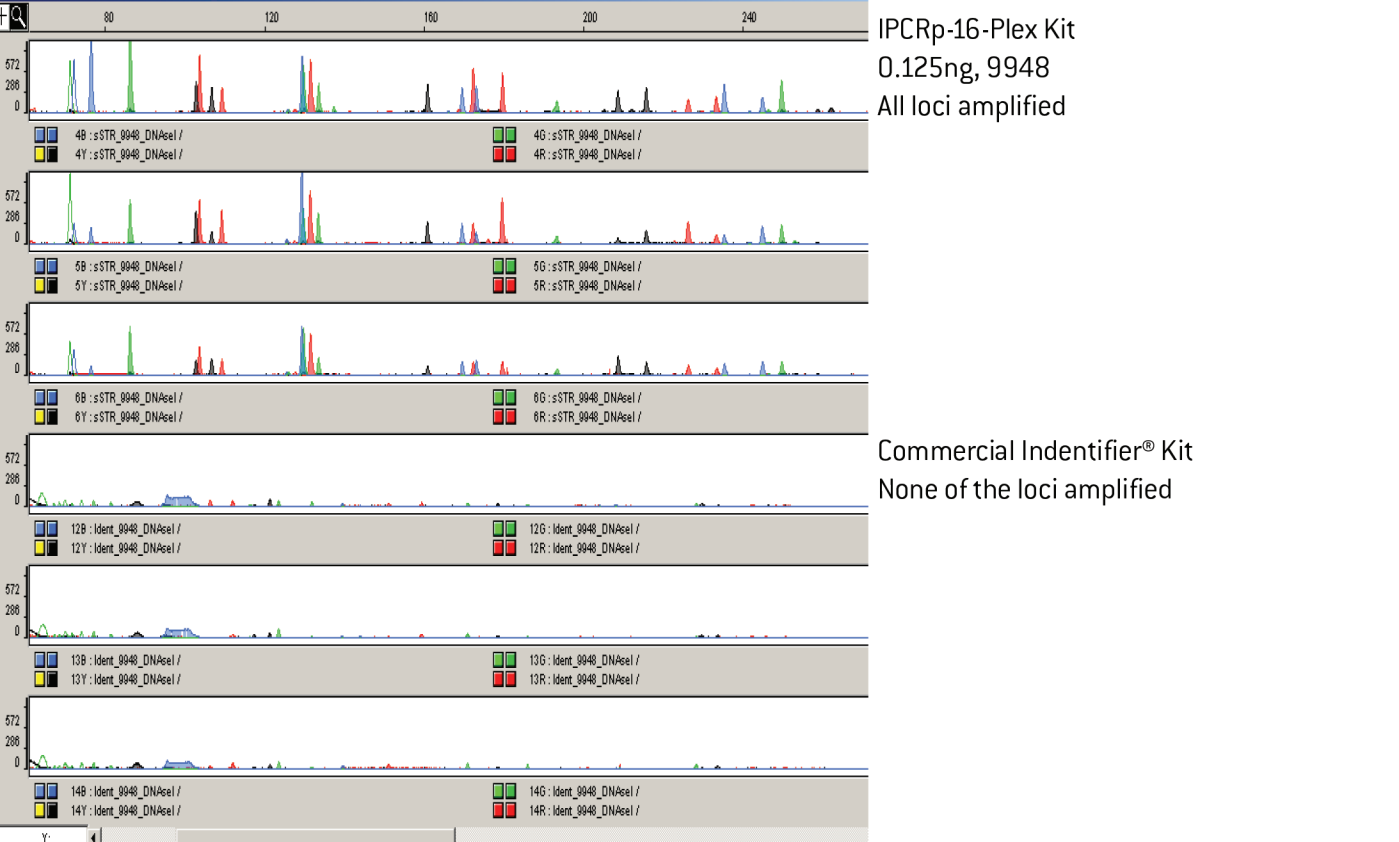
Amplification of degraded DNA, isolation of polymerase chain reaction products (IPCRp) 16-plex vs Identifiler® Kit.

The IPCRp method was also used by Yeung et al (21) and found to be increasing the signal strength of up to 17-fold compared to the classical PCR amplification, unfortunately, misquoting the original article of this powerful PCR amplification method and unfoundedly claiming originality. The IPCRp method was developed by Dimsoski and Woo at the Applied Biosystems precisely to address the issue of amplification of the low template DNA and degraded DNA and was first published in 2005 (4). The primary motivation for undertaking such a research project was the inability of the forensic community to efficiently genotype degraded samples collected from the site of the 9/11 mass disaster. Initial trials showed method performance that by far exceeded the commercially available products at a time. For example, when amplifying the fully degraded DNA with the IPCRp-modified Identifiler® kit all 16 loci became visible after being run on the genotyper instrument while none of the loci of the same degraded DNA could be amplified by the commercial Identifiler® kit (Figure 7).

Recent studies reported improvements in specific quantification of DNA extracted from animal feces (22) and improvements in DNA extraction methods (23-25) that together may improve chances for obtaining genotype from DNA extracted from feces. Another approach in improving the genotyping of challenging samples such as feces would be to apply sensitive PCR amplification method. In this study, because of its success with amplification of a low copy number and degraded DNA, the IPCRp method was used for genotyping equine fecal matter. Inherently, even the most sensitive PCR amplification method is dependent on the quality of the extracted DNA. Although, in this study specific PCR amplification method in combination with the specific extraction method improved possibility in obtaining genotypes from horse feces, the conclusion of this study are limited to the source of DNA, and not necessarily the same improvements should be expected in other situations, especially when one deals with not only degraded but also with inhibited DNA, that often is the case with field samples. The IPCRp method is recommended to be used on a variety of fecal sample types and could provide profiles from inhibited, low copy number, degraded DNA. Budowle and van Daal (26), in their landmark article on the directions for the future of the molecular biology, described a need for a method that would be “enabling the typing of samples of limited quantity and quality”. Considering the results of this and other studies, it can be concluded that the IPCRp is the method that can address the need for a typing samples of limited quantity and quality.
